# Antecedents of innovative behavior in public organizations: the role of public service motivation, organizational commitment, and perceived innovative culture

**DOI:** 10.3389/fpsyg.2024.1378217

**Published:** 2024-05-30

**Authors:** Geon Lee, Chulwoo Kim

**Affiliations:** ^1^Department of Public Administration, Hanyang University, Seoul, Republic of Korea; ^2^Department of Public Administration, Gachon University, Seongnam, Republic of Korea

**Keywords:** public service motivation, innovative behavior, organizational commitment, perceived innovative culture, innovative work behavior

## Abstract

**Introduction:**

This study examines the dynamics of public service motivation (PSM), organizational commitment, and perceived innovative culture and their collective influence on innovative behavior in public organizations. It uniquely focuses on intrinsic motivational factors, extends the scope of motivational studies to the public sector, and highlights the crucial role of organizational culture in fostering innovation.

**Methods:**

A web-based survey was administered to 1,021 public servants in the central government of the Republic of Korea. Structured questionnaires were used to collect data, and structural equation modeling (SEM) was employed to analyze the relationships between the variables.

**Results:**

The SEM results confirmed positive correlations between PSM and both organizational commitment and innovative behavior. However, contrary to expectations, organizational commitment did not significantly predict innovative behavior. Additionally, no mediating effect of organizational commitment was observed. Notably, perceived innovative culture was found to moderate the relationship between PSM and organizational commitment, and between organizational commitment and innovative behavior, particularly in environments with a strong innovation focus.

**Discussion:**

These findings underscore the significance of PSM in spurring innovative behavior in the public sector, broadening our understanding of intrinsic motivation. This study also accentuates the influence of organizational culture on these dynamics. In practical terms, this suggests the importance of nurturing individuals with high PSM and fostering an environment that balances perceived innovative culture. While contributing to the fields of organizational psychology and public administration, this study has certain limitations and indicates the need for further research in various contexts.

## 1 Introduction

The rapidly evolving global landscape, characterized by technological innovation, demographic shifts, and changing societal expectations, presents unique challenges and opportunities for public organizations (Northcott and Taulapapa, 2012; Hansen and Pihl-Thingvad, 2019; Nguyen et al., 2023). More specifically, innovations in digital public services are vital for addressing social and economic inequalities and ensuring universal access to services (Osborne and Brown, 2011; Bertot et al., 2016; Khorakian et al., 2019). Challenges such as climate change, public health crises, and social inequality further necessitate creative and innovative solutions beyond traditional bureaucratic approaches (Bernier et al., 2015; Brunetto et al., 2021). Moreover, periods of economic constraint and increasing demands for accountability and transparency compel public organizations to find innovative ways to maintain or enhance service delivery (Moore and Hartley, 2008; Agostino et al., 2020; Heinonen and Strandvik, 2020).

Therefore, the agility and adaptability of these institutions are paramount for addressing these complexities. Central to this adaptability is the innovative behavior of individuals within these organizations (Bysted and Hansen, 2015). To make an organization more efficient, innovative behavior has become important not only in the private sector but also in the public sector (Park and Jo, 2018). Innovative behavior can be a strategy for long-term organizational survival in response to rapidly changing internal and external environments. Public sector innovation, traditionally perceived as conservative and procedurally rigid, is not merely a function of technological adoption or procedural overhaul, but represents a profound shift in organizational ethos and behavior, encompassing a holistic approach to service design, delivery, and policy development (Miao et al., 2018). This necessitates a re-evaluation of the factors that drive innovative behavior in public sector employees (Zandberg and Morales, 2019), particularly focusing on psychological constructs.

Innovative behavior in the public sector is influenced by a constellation of factors, with recent literature underscoring the critical roles of leadership styles, public service motivation (PSM), organizational culture, psychological empowerment, learning orientation, and both intrinsic and extrinsic motivations (Gu et al., 2017; Klaeijsen et al., 2018; Ritala et al., 2019; Venketsamy and Lew, 2024). Transformational leadership, emphasizing inspirational motivation and intellectual stimulation, is pivotal in fostering an innovative environment (Askaripoor et al., 2020; Mahmood et al., 2020). PSM, the intrinsic desire to serve the public good, directly drives innovative efforts aimed at enhancing public welfare (Miao et al., 2018; Khan and Burdey, 2021). An organizational culture that prioritizes innovation, coupled with a climate that supports risk-taking and values flexibility, further catalyzes innovative behaviors (Nguyen et al., 2023). Psychological empowerment, reflecting employees’ perceptions of autonomy and significance (Miao et al., 2018), along with a strong learning orientation within the organization (Nguyen et al., 2023), are also instrumental in promoting innovation. Moreover, the balance between intrinsic motivation, derived from the joy of work itself, and extrinsic motivation, influenced by rewards and recognition (Liu et al., 2016; Fischer et al., 2019), plays a significant role in encouraging innovative behavior.

Recent studies have shed light on the relationship between PSM and innovative behavior in the public sector, highlighting the importance of mediating factors and the influence of leadership and cultural context. Miao et al. (2018) found that psychological empowerment mediates the relationship between PSM and innovative behavior in China, suggesting that empowerment is crucial for converting PSM into innovative actions. Askaripoor et al. (2020) in Iran and Mahmood et al. (2020) in Pakistan both emphasized the significant role of leadership in enhancing the PSM-innovation link. Khan and Burdey (2021) established a direct positive association between PSM and innovative behavior in Pakistan, a finding echoed by Vuong (2023) who also highlighted how leadership strengthens the PSM-innovation relationship. Suryani et al. (2023) and Rafique et al. (2023) further confirmed the positive impact of PSM on innovation in Pakistan, with Rafique et al. pointing out specific PSM dimensions like compassion and self-sacrifice as key drivers. Lastly, Nguyen et al. (2023) identified learning goal orientation as a mediator in Vietnam, underlining the role of a learning mindset in facilitating innovation through PSM. These studies collectively illustrate PSM’s pivotal role in driving public sector innovation, mediated by empowerment, leadership, and a culture of continuous learning.

The significance of innovative behaviors in driving administrative reform within public organizations is widely acknowledged. However, there is a shortage of research delving into the link between PSM and innovative behaviors, with a consideration of organizational culture. The limited empirical studies available are constrained by their focus on the cultural context of a specific country (i.e., Pakistan) as shown in Table 1. This study endeavors to address this gap by meticulously observing empirical data to elucidate the intricate relationship between PSM and the inclination toward innovative conduct, taking into account organizational culture within the Korean context.

**Table 1:** Recent studies addressing the relationship between PSM and innovative behavior.

Authors	Year	Relationship	Main findings
Miao et al.	2018	Indirect	Psychological empowerment mediates the relationship between PSM and innovative behavior of bureau directors in China.
Askaripoor et al.	2020	Mediation	PSM mediates between leadership and innovative work behavior in the public sector in Iran.
Mahmood et al.	2020	Mediation	PSM mediates between transformational leadership and innovative behavior of teachers in Pakistan.
Khan and Burdey	2021	Direct	PSM is positively associated with innovative behavior in Pakistan.
Vuong	2023	Direct and Moderation	PSM positively affected innovative work behavior and strengthens the relationship between leadership and innovative behavior in the public sector in Pakistan.
Suryani et al.	2023	Direct	PSM positively affect innovative behavior of civil servants in Pakistan.
Rafique et al.	2023	Direct	Attraction to policymaking, compassion, and self-sacrifice are associated with the innovative behaviors of educators in Pakistan.
Nguyen et al.	2023	Indirect	Learning goal orientation mediates between PSM and innovative behaviors among public sector employees in Vietnam.

Organizational commitment, the psychological attachment and loyalty employees feel toward their organization, has emerged as a potential mediator between public officials’ PSM and its positive outcomes (Vandenabeele, 2009; Im et al., 2016). When public officials are deeply committed to their organization, they are more likely to channel their altruistic motivations toward initiatives that not only align with the organization’s goals but also push the boundaries of traditional public service methods. Moreover, an perceived innovative culture characterized by an emphasis on creativity, openness to new ideas, and support for risk-taking may amplify or attenuate the effects of PSM and organizational commitment on innovative behavior (Austen and Zacny, 2015).

As a result, the literature points to a critical need for further exploration into the mechanisms through which PSM influences innovative behavior, with specific attention to the roles of organizational commitment and culture. This study aims to address these gaps by examining the mediating effect of organizational commitment and the moderating role of perceived innovative culture on the PSM-innovation nexus within public organizations.

This research provides significant theoretical contributions to the literature on PSM, organizational behavior, and innovation within public organizations. Our study makes three primary theoretical advancements:

First, it deepens the PSM literature by elucidating the direct influence of PSM on innovative behavior in the public sector. Unlike previous studies that have predominantly focused on PSM’s impact on job satisfaction and organizational commitment, our research explores its role as a catalyst for innovation, revealing how intrinsic motivation tied to public service can drive employees toward innovative behavior. This expands the understanding of PSM beyond traditional outcome variables, highlighting its critical role in fostering a culture of innovation in public organizations.

Second, our findings contribute to the organizational commitment literature by dissecting the mediating role of organizational commitment in the PSM-innovation nexus. By delineating the conditions under which organizational commitment acts as a bridge between PSM and innovative behavior, this study adds complexity to our grasp of commitment dynamics. This insight is particularly valuable for crafting targeted strategies that leverage organizational commitment to stimulate innovation, offering a refined perspective on managing employee engagement in the public sector.

**Figure 1:**
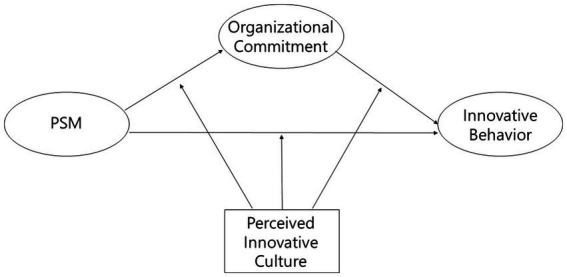
Conceptual model.

Lastly, by examining the moderating effect of perceived innovative culture, this study enriches the organizational culture literature. We demonstrate how the presence of an perceived innovative culture can amplify or mitigate the effects of PSM and organizational commitment on innovative behavior. This underscores the critical importance of aligning organizational culture with employee motivations to enhance innovative outcomes, offering a novel viewpoint on the strategic role of culture in driving public sector innovation. Overall, we propose the conceptual model in Figure 1.

## 2 Theoretical background and research hypotheses

### 2.1 Public service motivation

Although Rainey (1982) first coined the term “PSM” in his article published in *American Review of Public Administration* in 1982, Perry and Wise (1990) elaborated the concept of PSM. Before PSM had been posited, it was widely accepted that bureaucrats or those who work in the government are utility maximizers pursuing selfish ends in their workplace, and their motivation is the same as that of those who act in the market. Kelman (1987) was critical of this trend, stating that “*this account of the operation of the political process is a terrible caricature of reality. It ignores the ability of ideas to defeat interests, and the role of that public spirit plays in motivating the behavior of participants in the political process*” (p. 81). The origins of such positive views of a bureaucrat can be traced to the traditional intellectual approach to bureaucracy initiated by Max Weber. He envisaged those who work in the bureaucratic system (or bureaucrats) as sincere servants, suggesting that the bureaucrat is an impartial implementer in pursuit of organizational goals who does not place his personal goals over organizational goals in a bureaucratic system, even if his claims were empirically unproven.

For more than four decades, public administration scholars have identified differences between public and private organizations in terms of the behaviors and work-related attitudes of organizational members and organizational characteristics (Bozeman, 1987; Scott and Falcone, 1998; Rainey and Bozeman, 2000). Research on public-private sector differences has yielded fruitful empirical results, showing that the characteristics and behaviors of public organizations are sharply distinct from those of their private counterparts (Wright and Grant, 2010; Ingrams, 2020). One strand of these findings is that employees in public organizations (or bureaucrats) have different motivations and reward preferences from those in private organizations; numerous studies have reported identical findings. Rainey (1982), for example, compared middle managers in the public and private sectors, revealing that while the latter place a high importance on “higher pay” and “making a good deal of money,” the former focus on “engaging in meaningful public service” and “doing work that is helpful to other people” (p. 292). Based on these findings, he suggests developing the concept of PSM in research on public administration.

Full-scale PSM research has been conducted since the work of Perry and Wise (1990), who define PSM as “an individual’s predisposition to respond to motives grounded primarily or uniquely in public institutions and organizations” (p. 368). Perry and Wise propose three types of motives that lay the foundation for PSM: affective, norm-based, and rational. The affective motive refers to actions grounded in emotional responses to various social situations, the norm-based motive involves actions caused by endeavors to conform to social norms or rules, and the rational motive refers to behavior based on an individual’s desire to participate in policymaking. To measure the construct of PSM at the empirical level, Perry (1996) suggested four dimensions–public policymaking, public interest, compassion, and self-sacrifice–with 24 measurement items through survey-based observation of graduate students. Since the development of Perry’s PSM measurement items, ample empirical findings have been produced, constituting the bulk of evidence in the field.

The concept of PSM has two important implications for academia. First, there are two types of motivations–intrinsic versus extrinsic––in organization theory, which do not reflect or account for individuals’ motivation to serve the public. Thus, PSM fills this knowledge gap. Second, self-interest was recognized as a universal motivation of bureaucrats at a time before the concept of PSM when public choice theory dominated the territories of knowledge.

PSM has been studied extensively in the context of public administration and organizational behavior, with research highlighting its positive impacts on job satisfaction, commitment, and performance (Perry and Vandenabeele, 2015; Ritz et al., 2016).

Table 2 provides a synthesis of recent empirical investigations concerning the correlations between PSM and positive outcomes in organizational settings. Despite the multitude of empirical inquiries conducted thus far, they can be classified into several thematic clusters regarding these favorable outcomes. Chief among these outcomes are job attitudes, with job satisfaction and turnover intentions being notably significant in organizational contexts (Bright, 2020). High levels of PSM among public servants have been consistently linked to heightened job satisfaction during task performance (Naff and Crum, 1999; Andersen and Kjeldsen, 2013; Roh et al., 2016; Prysmakova and Vandenabeele, 2020; Zhang, 2023), consequently mitigating turnover intentions (Naff and Crum, 1999; Shim et al., 2017; Gan et al., 2020; Wang et al., 2024).

**Table 2:** Examples of the relationship between PSM and positive outcomes in pubic organizations.

Authors	Year	Positive outcomes	Main findings
Carpenter et al.	2012	Attraction to the public sector	Individuals with higher PSM levels are more likely to choose the public sector as their job preference.
Belle	2013	Job performance	PSM has been found to be positively associated with individual job performance among Italian nurses.
Anderson et al.	2014	Student performance	Students with higher PSM achieved academic performance.
Chen and Hsieh	2015	Knowledge sharing	There is positive association between PSM and knowledge sharing.
Koumenta	2015	Organizational citizenship behavior	PSM positively influences organizational citizenship behavior in UK government agencies.
Esteve et al.	2015	Collaborative behavior	PSM is positively associated with collaborative behavior in an organization.
Stazyk and Davis	2015	Ethical obligations	PSM is positively correlated with ethical obligations.
Cho and Song	2015	Whistleblowing	PSM increases public managers’ whistleblowing intention in U.S. federal government agencies.
Roh et al.	2016	Job satisfaction	Employees with high levels of commitment to public interest have high levels of personal satisfaction in an organization.
Campbell and Im	2016	Turnover intention	PSM has been linked to reduced turnover intention in 16 central government ministry headquarters in Korea.
Tepe	2016	Trust behavior	PSM is positively associated with trust behavior among college students.
Esteve et al.	2016	Prosocial behavior	PSM is positively related correlated with prosocial behavior among college students in the Netherlands.
Cooke et al.	2019	Work engagement	Positive relationship between PSM and work engagement has been found.
Yudiatmaja	2019	Service orientation	PSM positively influences employee service orientation.
Asseburg and Homberg	2020	Public sector choice	Individuals with higher PSM levels tend to choose a public sector job.
Sun	2021	Affective commitment	PSM is positively related to affective commitment to change in Chinese government agencies.
Ki	2021	Willingness to learn in an organization	PSM is positively associated with government officials’ willingness to learn.
Leisink et al.	2021	Volunteering activities	PSM positively affects employees’ participation in voluntary activities.
Weißmüller et al.	2021	Prosocial rule-breaking behavior	High-PSM individuals tend to engage in prosocial rule-breaking behavior within an organization.
Lim et al.	2022	Organizational performance	Public employees with higher PSM levels are more likely to contribute to organizational performance.
Park and Lee	2022	Morale	The higher the level of PSM, the higher the level of moral.
Gams-Morse et al.	2022	Anti-corruption, Bribe, cheat rates	Higher levels of PSM are associated with lower rates of anti-corruption, bribery, and cheating.
Nguyen et al.	2023	Learning goal orientation	Employees’ PSM is positively associated with employees’ learning goal orientation in Vietnamese government agencies.
Ripoll et al.	2023	Ethical behavior	Highly public service-motivated individuals tend to behave ethically.

PSM fosters socially desirable conduct both within and beyond organizational boundaries, stemming from individuals’ altruistic inclinations and commitment to public goods (Walton et al., 2017). Houston (2006) observed a higher propensity for charitable volunteering and blood donation among government agency employees, attributed to elevated levels of PSM compare to their private sector counterparts. Subsequent empirical studies have explored the association between PSM and volunteering behaviors, consistently affirming his relationship (Walton et al., 2017; Leisink et al., 2021).

Furthermore, PSM positively influences altruistic behaviors within organizations, exemplified by organizational citizenship behaviors which surpass formal role expectations, thereby enhancing organizational effectiveness and performance (Kim, 2006). Notably, a robust correlation between PSM and organizational citizenship behaviors has been observed in public administration studies. For instance, Koumenta (2015) demonstrated a significant promotion of organizational citizenship behaviors by PSM among British civil servants, concurrently reducing organizational deviance. Various empirical inquiries have replicated and reinforced these findings (Campbell and Im, 2016; Shim and Faerman, 2017; Abdelmotaleb and Saha, 2019; Ingrams, 2020; Li and Wang, 2022). Additionally, PSM serves as a precursor to prosocial and altruistic behaviors beyond organizational citizenship behaviors (Esteve et al., 2016; Piatak and Holt, 2020; Gans-Morse et al., 2022).

PSM is also under scrutiny as a driver of ethical conduct such as anti-corruption measures and whistleblowing within organizations. Despite conceptual overlaps with ethics, empirical studies have discerned a distinct positive association between PSM and ethical outcomes (Choi, 2004; Kwon, 2014; Stazyk and Davis, 2015; Wright et al., 2016). Particularly, whistleblowing, an ethical imperative in combating corruption and misconduct, is notably influenced by PSM (Cho and Song, 2015; Potipiroon and Wongpreedee, 2021; Prysmakova and Evans, 2022).

Lastly, research on PSM has explored its impact on individual and organizational performance within diverse settings. PSM emerges as a significant determinant of performance across various domains. Drawing on the field experimental method, Bellé (2013), for instance, showed nurses with higher levels of PSM achieved higher job performance in hospitals. Andersen et al. (2014) revealed that the students taught by teachers with high level of PSM exhibited higher academic achievement in school. Numerous studies affirm PSM’s role as a performance determinant (Naff and Crum, 1999; Ritz, 2009; Vandenabeele, 2009; Zhu and Wu, 2016; Lim et al., 2022; Thuy and Phinaitrup, 2023).

### 2.2 Innovative behavior and public service motivation

Janssen (2000) defined innovative behavior as a complex behavior that includes the generation, promotion, and idealization of ideas, specifically focusing on the individual creation of novel and useful ideas in the workplace across various domains. Innovation goes beyond performing tasks in a routine manner according to standard business procedures. It is an activity that voluntarily improves work methods, incorporates new technologies, views phenomena from a different perspective, and attempts to solve problems through new ideas (Woods et al., 2018). Furthermore, it is a continuous effort to spread experience and change fundamental work methods and systems (Scott and Bruce, 1994).

Innovative behavior is regarded as an important form of capital that enables an organization to effectively achieve its goals (Kanter, 1983; West and Far, 1990; Yuan and Woodman, 2010). In general, public organizations are not considered innovative because they do not operate within a market mechanism and are characterized by excessive rules and controls that constrain the innovative behavior of public servants (Miao et al., 2018). However, there has been recent emphasis on innovation that aims to improve efficiency and performance in the public sector (Fernandez and Moldogaziev, 2013; Waheed et al., 2017; Park and Yun, 2022).

While various factors, including organizational culture and climate, influence an individual’s innovation behavior, the psychological aspect of intrinsic motivation has been emphasized as an antecedent of innovative behavior (Yuan and Woodman, 2010). Given that innovation involves changing both the way people do things and the processes themselves, public sector employees are agents who facilitate and implement the innovative process (Nguyen et al., 2023). In public organizations, the outcome of innovation is an increase in the quality of public services, which benefits citizens. Public servants’ motivation to enhance the quality of their lives is a precondition for innovative behavior in public organizations.

The positive impact of intrinsic motivation on innovative or creative behavior has been well-documented in organizational studies (Liu et al., 2016; Fischer et al., 2019). Intrinsically motivated individuals tend to be curious, which facilitates creative ideas when they are at work (Devloo et al., 2015). In addition to intrinsic motivation, extrinsic motivation contributes to innovative or creative performance in organizations (Chen et al., 2010). For instance, extrinsic or monetary rewards are widely utilized as incentives for employees to promote innovative behavior in private-sector organizations.

As a type of motivation, PSM is understudied in the psychology or management domains, but it is regarded as a driving force that enables individuals to produce positive outcomes in public-sector organizations (Perry and Vandenabeele, 2015; Ritz et al., 2016). PSM is an important psychological resource that drives innovative behavior in public organizations, and it is an important factor in organizational innovative behavior because members of public organizations are motivated by the ideal of serving the public interest and are committed to serving the people rather than monetary rewards. One of the most important factors in innovative behavior is the voluntary participation of organizational members. This indicates that innovative behavior is driven by individuals’ attitudes, psychology, and motivation, which are intrinsic to the organization, rather than by external rewards and control.

Perry and Wise (1990) propose that PSM encompasses three distinct motives: rational, normative, and affective. The rational motive entails individuals’ aspiration to engage in the policymaking process, prioritizing societal or communal interests over personal gain, contrary to the assumptions of economic theory. Normative motive involves a desire to advance the common good and fulfill civic duties as a citizen. Affective motive reflects a commitment to government programs driven by a conviction of their societal significance, accompanied by feelings of empathy and affection toward others. These three motives, integral to PSM, are closely linked to intrinsic motivation (Jung et al., 2018).

The measurement of PSM relies on four sub-elements that mirror these motives. Rational motive is gaged by the inclination to participate in policymaking and attraction toward attraction to policymaking (APM), while normative motive is assessed through commitment to public interest (CPI), indicating the extent to which individuals strive for the common good. Affective motive is measured through compassion (COM) and self-sacrifice (SS) (Perry, 2000). Since the sub-dimensions of PSM overlap with characteristics of intrinsic motivation, some scholars contend that PSM constitutes a form of intrinsic motivation (e.g., Crewson, 1997; Houston, 2000).

Despite similarities, some scholars distinguish between PSM and intrinsic motivation (Paarlberg and Lavigna, 2009; Bozeman and Su, 2015). PSM tends to be more altruistic, driving individuals to perform challenging tasks not solely for personal enjoyment but for the benefit of society and communities. Consequently, individuals with high PSM levels are inclined to invest extra effort in achieving outcomes that hold meaning and significance for society and others (Perry and Hondeghem, 2008; Ki, 2021). Rafique et al. (2023) identified correlations between PSM sub-dimensions and innovative behaviors within the Pakistani context. Thus, public service-motivated individuals consistently approach problem-solving from a citizen’s perspective, striving to ensure that public services are delivered in an innovative manner that is both convenient and efficient for citizens.

In addition, innovative behavior can be explained by PSM because this type of motivation is based on self-sacrifice, aims to realize the public good, and drives proactive and active behavior. Given that innovative behavior involves trying to solve problems through new ideas beyond routine work procedures and taking the risk of failure, it is difficult to achieve without an active and positive psychological state and motivation to accept the risk of failure. Therefore, PSM serves as a key driver of innovative behavior because individuals motivated by public service values are more likely to engage in activities that foster organizational innovation.

While the nexus between PSM and innovative behavior has not been comprehensively explored, recent studies have suggested a significant positive relationship. Employees with high PSM may be more inclined to engage in innovative behaviors, as they align with their intrinsic motivation to serve public goods and improve public welfare (Miao et al., 2018; Lee et al., 2020). For instance, Vuong (2023) found that civil servants with high levels of PSM tended to exhibit innovative work behavior in Vietnamese local governments. Khan and Burdey (2021) and Suryani et al. (2023) discovered that individuals motivated by public service are inclined to exhibit innovative behaviors in the workplaces in Pakistan.

Self-determination theory (SDT), proposed by Deci and Ryan (1985), explains PSM can be a potent intrinsic motivator for individuals in the public sector. PSM, characterized by an altruistic desire to serve the public and contribute to society, aligns with the principles of SDT (autonomy, competence, and relatedness). Specifically, PSM fulfills the need for autonomy (engaging in work that feels personally meaningful), competence (feeling effective in contributing to public good), and relatedness (connecting with societal values and the community) (Corduneanu et al., 2020). These alignments suggest that civil servants with high PSM are motivated to serve the public good. Therefore, they will likely engage in proactive behaviors, seeking new and creative work processes to deliver public service efficiently and effectively to citizens.

*H1*: Public service motivation is positively related to innovative behavior.

### 2.3 Mediating effect of organizational commitment

Organizational commitment, as defined by Meyer and Allen (1991), encompasses an employee’s psychological attachment and loyalty toward their organization, highlighting its complex and multifaceted nature. It encompasses affective commitment (emotional attachment to the organization), continuance commitment (perceived cost of leaving the organization), and normative commitment (sense of obligation to remain with the organization). Although organizational commitment is a multidimensional construct, these three dimensions are not mutually exclusive (Camilleri and Van Der Heijden, 2007).

While PSM and organizational commitment exhibit mutual interdependence, it is more logically argued that PSM serves as an antecedent of organizational commitment within public organizations (Vandenabeele, 2009). As previously noted, there are three dimensions comprising the concept of organizational commitment. Normative commitment pertains to an obligation-based loyalty to the organization (Allen and Meyer, 1990). Public service-motivated individuals entering and working in the public sector strongly embrace the values and goals of public service for the betterment of the public good (Witesman and Walters, 2013). The alignment between individuals and the organization in public services enables employees to uphold the obligations expected by the organization. Adherence to norms and values can serve as a fulfilling motivation for public servants (Andersen et al., 2013). Normative commitment acts as a cohesive force between public service-motivated employees and value-oriented behaviors within public organizations. Consequently, public employees, guided by normative and value-oriented attitudes, endeavor to enhance society by refining and improving the process through which public services are delivered.

Affective commitment refers to emotional attachment to the organization, where organizational members identify themselves with the organization, leading to enjoyment of tasks within the organization (Allen and Meyer, 1990). Public service-motivate individuals identify themselves with public services, fostering a public service identity (Bednarczuk, 2018). Individuals with a high level of affective commitment, or public service identification, tend to derive satisfaction from their tasks and actively engage in them, thereby enhancing organizational performance. Increased identification with the public service organization correlates with a higher likelihood of employees making significant personal investments in the organization and actively engaging in actions that contribute positively to its success (Miao et al., 2019).

Continuance commitment can be defined as “a desire to maintain organizational membership” (Porter et al., 1974:604). People with this type of commitment and PSM typically internalize the values of public services through the socialization process during their tenure in public organizations. Empirical evidence supports that organizational members with a high level of PSM exhibit a low intention to turnover (Campbell and Im, 2016; Shim et al., 2017; Jia et al., 2022). In other words, low turnover intention indicates high continuance commitment. As a result, employees with high continuance commitment are presumed to engage in innovative behaviors to address imminent challenges, staying with the organization to contribute to its performance and sustainability.

According to social exchange theory, relationships within organizations are driven by the reciprocal exchange of resources, benefits, and rewards between individuals and the organization (Cropanzano and Mitchell, 2005; Blau, 2017). Employees with high levels of PSM are motivated by altruistic values and a desire to contribute to the public good. The initial step in this reciprocal relationship is catalyzed by employees’ perception that their altruistic efforts and motivations are recognized and supported by the organization. Next, committed employees are more likely to take initiative, propose new ideas, and implement changes that align with organizational goals, viewing innovation as a way to give back to the organization and further its mission. As a result, organizational commitment mediates the relationship between PSM and innovative behavior by acting as the mechanism through which the social exchange process translates intrinsic motivation (stemming from PSM) into actions that benefit the organization (innovative behavior). The stronger the commitment, the more likely employees are to engage in innovative activities, as they feel an emotional and psychological investment in the organization’s success (Mowday et al., 1979; Wright and Pandey, 2008). Specifically, Vandenabeele (2009) empirically found that PSM is linked to performance exhibiting a high correlation with innovative behavior mediated by organizational commitment.

Consequently, the positive influence of PSM on innovative behavior is mediated by the degree of an employee’s commitment to the organization, as stronger commitment may lead to greater involvement in innovative activities. Accordingly, we hypothesize that:

*H2*: Organizational commitment mediates the effect of public service motivation on innovative behavior.

### 2.4 Moderating effect of perceived innovative culture

Organizational culture refers to a “complex set of values, beliefs, assumptions, and symbols that define the way in which a firm conducts its business” (Barney, 1986, p. 657). Culture is a collective context, such as an institution, that guides organizational members’ behaviors and choices. Within this context, individuals internalize and learn shared cultural values to sustain in-group homogeneity (Wilkins and Ouchi, 1983; Büschgens et al., 2013). When innovation is a shared value within an organization, it fosters an perceived innovative culture and actively motivates its members to innovate in terms of both willingness and behavior.

The influence of organizational culture, specifically perceived innovative culture, on employee behavior is well-established in organizational psychology. An perceived innovative culture characterized by support for creativity, tolerance of risk, and openness to new ideas is considered crucial for fostering innovation within organizations (Martins and Terblanche, 2003). A group with a collective organizational culture of innovation will serve as a catalyst to enhance the level of innovative behavior and its relationship with other factors influencing innovation compared to a group lacking such a culture.

The moderating role of perceived innovative culture in the relationship between PSM, organizational commitment, and innovative behavior is rooted in fit theory (Kristof, 1996). According to this perspective, the congruence between an individual’s values (such as those associated with PSM) and the organizational environment (such as an perceived innovative culture) enhances the likelihood of certain behaviors, including innovation (Schneider, 1987; Chatman, 1989). Thus, perceived innovative culture may amplify or mitigate the effects of PSM and organizational commitment on innovative behavior.

The alignment between PSM and an perceived innovative culture arises because individuals with a high level of PSM are often intrinsically motivated to achieve outcomes that benefit the public and seek out creative ways to overcome barriers to public service delivery. An organizational culture that values and supports innovation can amplify the impact of PSM by providing the resources, support, and recognition needed to transform creative ideas into tangible improvements in public services. Thus, when PSM and an innovative organizational culture coexist, the organization is more likely to foster a proactive and creative workforce dedicated to public service excellence.

Also, the link between organizational commitment and innovation behavior is grounded in the idea that committed employees are more likely to engage in behaviors that go beyond their basic job requirements, including innovative behavior. This is because committed employees have a stronger desire to contribute to the organization’s success and are more willing to engage in risk-taking and experimentation, which are essential for innovation. Furthermore, committed employees are likely to have a deeper understanding of the organization’s goals and challenges, enabling them to identify opportunities for innovation that align with organizational objectives.

Hence, the presence of an perceived innovative culture within an organization strengthens the relationship between PSM, organizational commitment, and innovative behavior, creating an environment more conducive to innovation.

Accordingly, Hypothesis 3-1 states that an perceived innovative culture influences how PSM translates into organizational commitment, potentially enhancing the alignment between personal values and organizational objectives. Hypothesis 3-2 implies that the impact of organizational commitment on innovative behavior varies depending on the level of perceived innovative culture, with a stronger culture likely to enhance the commitment-behavior link. Finally, hypothesis 3-3 suggests that perceived innovative culture within an organization can moderate the relationship between PSM and innovative behavior.

*H3*: Perceived innovative culture moderates the relationships among PSM, organizational commitment, and innovative behavior.

*H3-1*: Perceived innovative culture moderates the relationship between PSM and organizational commitment.

*H3-2*: Perceived innovative culture moderates the relationship between organizational commitment and innovative behavior.

*H3-3*: Perceived innovative culture moderates the relationship between PSM and innovative behavior.

## 3 Methods

### 3.1 Sampling and data collection

The survey in this study targeted public servants within the central government of the Republic of Korea. Due to the challenge of obtaining a comprehensive sampling frame of all government agency employees in Korea, we utilized two sources for sampling: an online panel pool and a list of employees from the Ministry of the Interior and Safety (MOIS). The online panel pool, owned by Mbrane Public, a reputable research company in Korea, comprises approximately 1.5 million individuals as of May 2022. We distributed an email containing a web survey instrument and sent text messages requesting participation to all 6,333 panelists who identified their occupation as “public servant in a centralized administrative organization.” Ultimately, 714 respondents out of the 6,333 panelists participated in the web survey, resulting in a survey participation rate of 11.3%.

However, due to the insufficient sample size to meet our target, we extended the survey to include public employees in the central government through the MOIS. A cooperation letter containing the survey URL was issued to 3,845 employees working in the MOIS headquarters and agencies, with 307 employees participating in the survey, yielding a response rate of 8.0%. The combined response rate for both surveys was 10.0%. Data collection took place from May 24 to June 5, 2022, and was conducted by Mbrane Public. To analyze the survey data and test the proposed hypotheses, structural equation modeling (SEM) was employed.

### 3.2 Measures

#### 3.2.1 Public service motivation

PSM was measured using four survey items from PSM measurement instruments suggested by Kim (2009). The four items include: ‘Meaningful public service is very important to me’ (Commitment to the public interest dimension), ‘I am interested in making public programs that are beneficial for my country I belong to’ (Attraction to policymaking dimension), ‘I feel sympathetic to the plight of the underprivileged’ (Compassion dimension), and ‘I am prepared to make enormous sacrifices for the good of society’ (Self-sacrifice dimension). The reliability of the survey items, as measured by Cronbach’s α, was 0.80.

#### 3.2.2 Perceived innovative culture

Perceived innovative culture was measured using two survey items modified from Cameron and Quinn’s (1999) Competing Values Framework Scale. The two survey items include ‘My organization emphasizes innovation and creativity,’ and ‘My organization takes into account employees’ insights to resolve the challenges’ The value of Cronbach’s α of the scale was 0.81. These two items were incorporated into a single metric variable. We transformed the variable into a non-metric variable and dichotomized it into high- and low-innovation culture groups based on the average value.

#### 3.2.3 Organizational commitment

Organizational commitment was evaluated using four modified survey items referenced from Allen and Meyer (1990). The four survey items are: “I feel a strong sense of belonging to my organization’ (Affective commitment dimension), I feel proud of belonging to my organization’ (Affective commitment dimension), ‘I am willing to work additionally if my organization wants’(Normative commitment dimension), and ‘I have never thought about leaving this organization’(Continuance commitment dimension). The reliability of the items in this study was indicated by a Cronbach’s α value of 0.81.

#### 3.2.4 Innovative behavior

Innovative behavior was assessed using three modified survey items developed by Scott and Bruce (1994). The three items include: ‘I frequently generate creative ideas,’ and ‘I try to develop new ideas to solve problems at work,’ ‘I do my best to revamp the irrational status quo.’ The Cronbach’s α value of the scale was 0.86.

#### 3.2.5 Control variables

We include employee gender (1: male, 0: female), education level (1: graduate degree, 0: undergraduate degree), and job tenure (0–38 years) as control variables that may affect innovative behavior in the model. All the variables except the control variables were scored on a five-point Likert scale. The sample consists of 562 males (55.0%) and 459 females (45.0%). In terms of education, 327 individuals are graduates (31.7%), and the majority, 697, have an undergraduate level of education or less (68.3%). Regarding job tenure, the sample is divided among those with less than 2 years (109 individuals, 10.7%), 2–6 years (276 individuals, 27.0%), 6–10 years (188 individuals, 18.4%), 11–20 years (261 individuals, 25.6%), and more than 20 years (187 individuals, 18.3%).

## 4 Analytical results

### 4.1 Reliability and validity

The factor loadings of the measurement items demonstrated sufficient representation of their respective constructs. Both the overall Cronbach’s α value and those of all constructs exceeded 0.8, exceeding the established threshold of 0.70 (Kline, 2016). Convergent and discriminant validity were assessed to ensure the convergence of multiple indicators within the same construct and the distinctiveness of the indicators across different constructs (Neuman, 2011). The evaluation of convergent validity relies on average variance extracted (AVE) and composite reliability (CR). As presented in Table 3, the CR values exceeded 0.8 and the AVE values exceeded 0.6, meeting the criteria for sufficient convergent validity.

**Table 3:** Reliability of the constructs.

Variable	Items	Factor loading	CR	AVE	Cronbach’s α
Public service motivation	PSM 1	0.786***	0.846	0.702	0.803
	PSM 2	0.730***			
	PSM 3	0.621***			
	PSM 4	0.708***			
Organizational commitment	OC 1	0.807***	0.871	0.748	0.814
	OC 2	0.658***			
	OC 3	0.803***			
	OC 4	0.668***			
Innovative behavior	IB 1	0.822***	0.861	0.790	0.861
	IB 2	0.864***			
	IB 3	0.778***			
Perceived innovative culture	PC 1	0.745***	0.824	0.809	0.814
	PC 2	0.923***			

***p < 0.001.

For discriminant validity, a comparison was made between correlation coefficients of the three latent variables and the square root values of their respective AVE. According to the established criterion, discriminant validity was affirmed if the square root values of AVE surpass the correlation coefficients. The results in Table 4 indicate that the square root values of the AVE for all four latent variables consistently exceeded the correlation coefficients among the variables, confirming the model’s satisfactory discriminant validity.

**Table 4:** Correlation coefficients and discriminant validity.

Variable	$\sqrt{AVE}$	PSM	OC	IB	PC	Gender	Work year	Edu
PSM	0.83	1						
OC	0.86	0.28***	1					
IB	0.88	0.24***	0.22***	1				
PC	0.89	0.33***	0.29***	0.17***	1			
Gender	–	0.15***	0.06***	0.08	0.01	1		
Work year	–	0.20***	1.36***	1.66	0.58*	1.18***	1	
Edu	–	0.07*	0.02	0.02	0.01	0.01	0.63***	1

*p < 0.05; ***p < 0.001.

### 4.2 Common method variance

To check for common method bias, we followed Podsakoff et al. (2003) and applied Harman’s one-factor technique. Harman’s one-factor analytical results identified the variance of the first factor as 38.44%, which was less than 50%, indicating that common-source bias was not a serious concern in our data. Additionally, the Heterotrait-Monotrait (HTMT) ratios for our study ranged from 0.33 to 0.598, effectively confirming the discriminant validity of the constructs. Since all HTMT values are significantly below the conventional thresholds of 0.85, it is clear that the constructs are distinct and measure separate phenomena (Henseler et al., 2015). This is a crucial validation point, particularly in addressing potential concerns related to common method variance.

### 4.3 Confirmatory factor analysis

Confirmatory factor analysis (CFA) was conducted to identify the optimal model through multiple confirmatory factor comparisons. As depicted in Table 5, the model fit least favorably when all factors were combined into a single factor ( $\chi^{2}=$ 2343.765, *df* = 65, CFI = 0.608, TLI = 0.530, RMSEA = 0.185, SRMR = 0.110). The most suitable model was determined to be the four-factor model, demonstrating a good fit to the data ( $\chi^{2}$ = 327.478, *df* = 59, CFI = 0.954, TLI = 0.939, RMSEA = 0.067, SRMR = 0.050). Significantly, the proposed four factor-model outperformed the three-factor model, as evidenced by Δ $\chi^{2}$ (3) = 485.146, *p* < 0.001.

**Table 5:** Confirmatory factor analysis.

Model	*χ* ^2^	*df*	CFI	TLI	RMSEA	SRMR
Four-factor model (PSM, PC, OC, IB)	327.478	59	0.954	0.939	0.067	0.050
Three-factor model (PSM, OC + PC, IB)	812.624	62	0.871	0.838	0.109	0.068
Three-factor model (PSM + PC, OC, IB)	975.656	62	0.843	0.802	0.120	0.081
Two-factor model (PSM + OC, PC + IB)	1,616.130	64	0.733	0.675	0.154	0.107
Two-factor model (PSM + PC, OC+ IB)	2,114.574	64	0.647	0.570	0.177	0.109
One-factor model (PSM + OC + IB + PC)	2,343.765	65	0.608	0.530	0.185	0.110

### 4.4 Hypothesis testing

#### 4.4.1 Main effects

**Figure 2:**
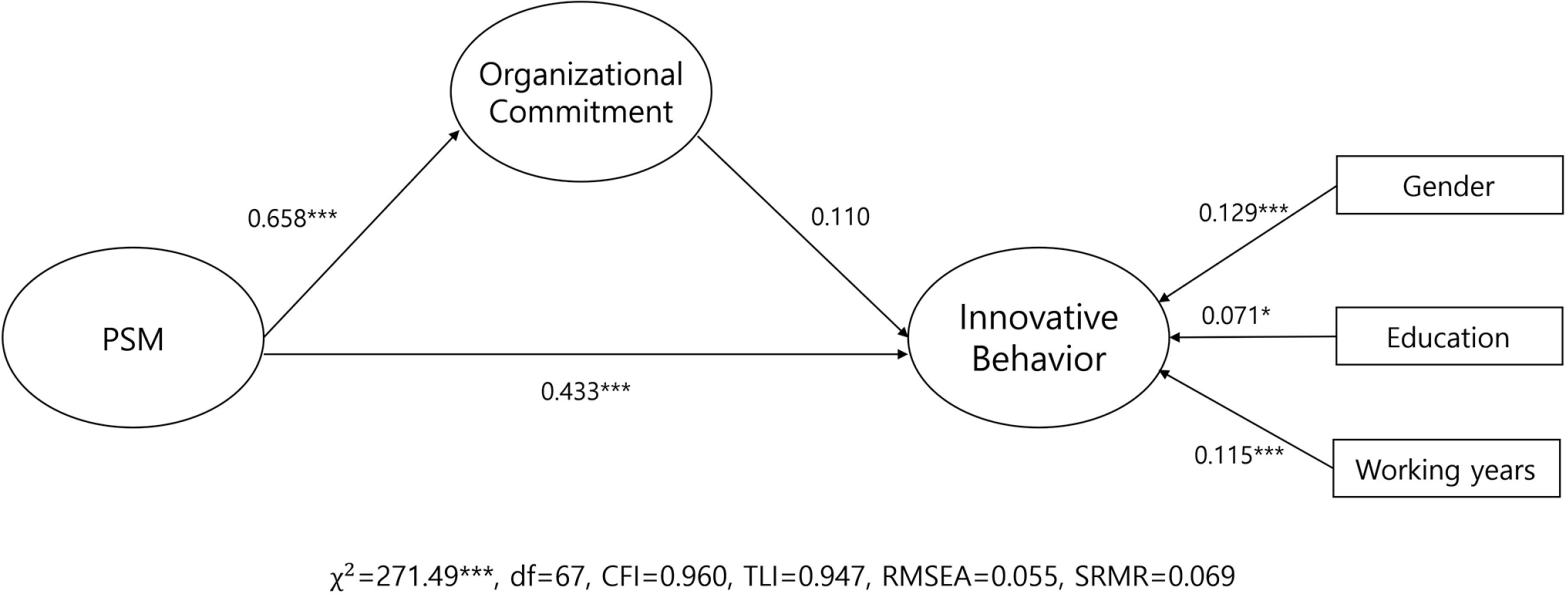
Path coefficients of the proposed model.

Structural equation modeling revealed a favorable fit to the data ( $\chi^{2}$ = 271.19, *df* = 67, CFI = 0.960, TLI = 0.947, RMSEA = 0.055, SRMR = 0.069). As illustrated in Figure 2 and Table 6, PSM exhibited a positive relationship with organizational commitment (*β* = 0.658, *p <* 0.001), and was also positively associated with innovative behavior (*β* = 0.433, *p <* 0.001), supporting our hypotheses. Contrary to our expectations, however, organizational commitment did not emerge as a predictor of innovative behavior (*β* = 0.110, *p* > 0.05).

**Table 6:** Main effects of the proposed model.

Direct Path	Coefficient	S.E	95% Confidence Interval	
			Lower 2.5%	Upper 2.5%
PSM → OC	0.658***	0.034	0.591	0.723
OC → IB	0.110	0.062	−0.012	0.234
PSM → IB	0.433***	0.061	0.312	0.549
Gender → IB	0.129***	0.031	0.068	0.188
Education → IB	0.071*	0.029	0.014	0.128
Working year → IB	0.115***	0.029	0.058	0.172

*p < 0.05; ***p < 0.001.

Regarding the control variables, gender demonstrated a positive association with innovative behavior, indicating that male employees were more inclined to be innovative than their female counterparts. Education was found to be linked to innovative behavior, suggesting that employees with graduate degrees are more likely to innovate than those with undergraduate degrees. In addition, job tenure displayed a positive association with innovative behavior in public organizations, which supports that Woods et al. (2018)‘s findings that employees’ organizational tenure has a pivotal role of innovative work behavior.

#### 4.4.2 Mediation effect

To examine the mediation effect, we performed bootstrap resampling with 5,000 replications. The bootstrap analysis in Table 7, revealed that the mediation effect value of organizational commitment between PSM and innovative behavior was 0.08, with a 95% confidence interval of [−0.009, 00172], which includes 0, indicating the absence of a mediating effect of organizational commitment in the model, contrary to our hypothesis. In this causal path, the direct effect was 0.48 and the indirect effect was 0.08, and the total effect was thus 0.56. This finding indicates that most of the effect can be attributed to the direct relationship between PSM and innovative behavior.

**Table 7:** Analytical results of bootstrap resampling for mediation (replication = 5,000).

Mediation	Estimate	S.E.	95% CI
PSM → OC → IB	0.080	0.045	−0.009, 0.172
Total effect	0.560	0.049	0.463, 0.656
Direct effect	0.480	0.072	0.339, 0.621
Indirect effect	0.080	0.045	−0.009, 0.172

#### 4.4.3 Moderating effects

To examine the moderating effect of perceived innovative culture, we conducted separate structural equation modeling for the high and low innovation groups. Table 8 presents the coefficients for all paths in both groups. The effect value of PSM on organizational commitment for the high innovation group was 0.832 (*p <* 0.001), while that for the low innovation group was 0.557 (*p <* 0.001). However, it remains uncertain whether the coefficient of the high innovation group differs significantly from that of the low innovation group.

**Table 8:** Group analysis for moderation effects.

Path	High innovation group	Low innovation group	Unconstrained model	Constrained model
	β (S.E.)	β (S.E.)	$\chi^{2}$ (*df*)	$\chi^{2}$ (*df*)
PSM → OC	0.832*** (0.060)	0.557*** (0.076)	361.66 (150)	373.27 (151)
OC → IB	0.219** (0.081)	−0.029 (0.065)	361.66 (150)	367.31 (151)
PSM → IB	0.334*** (0.094)	0.550*** (0.064)	361.66 (150)	365.17 (151)

**p < 0.01; ***p < 0.001.

To test the significance of the difference between the two coefficients, the unconstrained model was compared with the constrained model while holding the effects of PSM on organizational commitment equal. The chi-square difference value, $\Delta \chi^{2}\left(1\right),$ between the unconstrained ( $\chi^{2}$ = 361.66, *df* = 150) and constrained ( $\chi^{2}$ = 373.27, *df* = 151) models was 11.61, exceeding the critical value of 3.84 at the 0.05 significance level. This indicates a moderating effect, suggesting that the effect of PSM on organizational commitment is significantly higher for the high-innovation group than for the low-innovation group.

For the causal path between organizational commitment and innovative behavior, the effect of organizational commitment on innovative behavior for the high innovation culture group was positive and statistically significant (β = 0.219, *p <* 0.01), but that of its counterpart was not statistically significant. The chi-square difference value, $\Delta \chi^{2}\left(1\right),$ between the unconstrained ( $\chi^{2}$ = 361.66, *df =* 150) and constrained ( $\chi^{2}$ = 367.31, *df =* 151) models was 5.65, indicating that the effect for the high innovation group is significantly different from that for the low innovation group—that is, there is a moderating effect.

For the effect of PSM on innovative behavior, the chi-square difference value, $\Delta \chi^{2}\left(1\right),$ between the unconstrained and constrained models was 3.51, which is less than the critical value of 3.84. This finding demonstrates that perceived innovative culture has no moderating effect.

## 5 Discussion and implications

### 5.1 Discussion

The results offer significant insight into the dynamics of innovative behavior within public organizations. The acceptance of Hypothesis 1 confirms that PSM is positively related to innovative behavior. This finding aligns with the existing literature, suggesting that employees motivated by a desire to serve the public are more inclined to engage in innovative activities aimed at making citizens more comfortable with public services (Miao et al., 2018; Lee et al., 2020; Nguyen et al., 2023). This relationship underscores the importance of intrinsic motivation in fostering an environment conducive to innovation, particularly in the public sector.

The non-acceptance of Hypothesis 2 suggests that organizational commitment may not play the hypothesized mediating role in the relationship between PSM and innovative behavior. This could indicate that the direct influence of PSM on innovative behavior is not significantly channeled through organizational commitment. This finding suggests that PSM directly influences innovative behavior without mediation through organizational commitment, perhaps implying that the intrinsic motivation provided by PSM is sufficient to drive innovative behavior independently. Alternatively, the nature of public sector work, with its complex regulations and bureaucratic constraints, might limit the extent to which individual commitment can translate into observable innovative outcomes (Demircioglu and Audretsch, 2017; Acar et al., 2018).

The acceptance of Hypotheses 3–1 and 3–2 with positive moderation underscores the significant role of perceived innovative culture in enhancing the relationships between PSM and organizational commitment (H3-1) and between organizational commitment and innovative behavior (H3-2). This indicates that in environments where innovation is culturally valued and nurtured, employees with high PSM are likely to develop stronger organizational commitment, which in turn, more effectively translates into innovative behavior. This result highlights the pivotal role of an innovative organizational culture in leveraging employee motivation and commitment toward fostering innovation (Lukoto and Chan, 2016; Li and Liu, 2022; Pradana et al., 2022).

The finding that Hypothesis 3-3, positing that perceived innovative culture moderates the relationship between PSM and innovative behavior, is not supported prompts several possible explanations. This outcome suggests that the influence of an innovative organizational culture may not be as pivotal in moderating the impact of PSM on innovative behavior as initially theorized.

The results offer significant insight into the dynamics of innovative behavior within public organizations. The hypothesis 1 was supported. It confirms that PSM is positively related to innovative behavior. This finding aligns with the existing literature, suggesting that employees motivated by a desire to serve the public are more inclined to engage in innovative activities aimed at making citizens more comfortable with public services (Miao et al., 2018; Lee et al., 2020; Nguyen et al., 2023). This relationship underscores the importance of intrinsic motivation in fostering an environment conducive to innovation, particularly in the public sector.

The hypothesis 2 was not supported. It suggests that organizational commitment may not play the hypothesized mediating role in the relationship between PSM and innovative behavior. This could indicate that the direct influence of PSM on innovative behavior is not significantly channeled through organizational commitment. This finding suggests that PSM directly influences innovative behavior without mediation through organizational commitment, perhaps implying that the intrinsic motivation provided by PSM is sufficient to drive innovative behavior independently. Alternatively, the nature of public sector work, with its complex regulations and bureaucratic constraints, might limit the extent to which individual commitment can translate into observable innovative outcomes (Demircioglu and Audretsch, 2017; Acar et al., 2018). Given the limited research on this relationship, further investigation is necessary across different sociocultural and methodological contexts.

The hypotheses 3–1 and 3–2 were supported; positive moderation underscores the significant role of perceived innovative culture in enhancing the relationships between PSM and organizational commitment (H3-1) and between organizational commitment and innovative behavior (H3-2). This indicates that in environments where innovation is culturally valued and nurtured, employees with a high level of PSM are likely to develop stronger organizational commitment, which in turn, more effectively translates into innovative behavior. This result highlights the pivotal role of an innovative organizational culture in leveraging employee motivation and commitment toward fostering innovation (Lukoto and Chan, 2016; Li and Liu, 2022; Pradana et al., 2022).

The finding that hypothesis 3-3, positing that perceived innovative culture moderates the relationship between PSM and innovative behavior, is not supported prompts several possible explanations. This outcome suggests that the influence of an innovative organizational culture may not be as pivotal in moderating the impact of PSM on innovative behavior as initially theorized.

One possible explanation for this result could be the inherent strength of PSM itself. Individuals with high PSM may be intrinsically motivated to innovate, regardless of organizational culture. This aligns with one of the core principles of PSM that individuals are driven by a commitment to the public good and societal values, potentially overshadowing environmental factors like organizational culture (Perry and Vandenabeele, 2015).

### 5.2 Implications

#### 5.2.1 Theoretical implications

The study’s findings on the interplay between PSM, organizational commitment, innovative behavior, and the role of perceived innovative culture within public organizations offer several potential contributions to the literature of organizational psychology. These contributions revolve around understanding how individual motivations and organizational factors interact to influence behavior in workplace settings, particularly in the context of fostering innovation. Here are some possible contributions.

First, the direct relationship between PSM and innovative behavior is in line with self-determination theory, which emphasizes the importance of intrinsic motivation in driving workplace behavior (Klaeijsen et al., 2018; Ryan and Deci, 2020). This finding contributes to organizational psychology by providing empirical support for theories that emphasize the role of intrinsic factors, such as a sense of purpose or a desire to contribute to the public good, in motivating behavior beyond extrinsic rewards or formal organizational structures (Reiss, 2012; Kuvaas et al., 2017).

Second, the findings related to the moderating role of perceived innovative culture in enhancing the relationship between PSM and organizational commitment and between organizational commitment and innovative behavior contribute to organizational culture theories. They highlight the importance of alignment between individual motivations, organizational commitment, and the broader cultural context in facilitating innovative behaviors. This aligns with and expands upon person-organization fit theory, providing a view of how culture interacts with individual and organizational-level factors to support or hinder innovation (Goldberg et al., 2015; Lau et al., 2017; Chen et al., 2018).

Third, the study reveals a direct relationship between PSM and innovative behavior in public organizations, challenging the conventional roles of organizational commitment and culture as mediators and moderators. Organizational commitment is based on extrinsic rewards within an organization. However, since PSM is based on intrinsic rewards and operates through a passion for serving the public interest, it can lead to innovative behavior even in the absence of a strong connection to rewards, especially in public organizations. Therefore, self- determination theory can account for this relationship, suggesting that the dynamics of intrinsic motivation play a significant role. In conclusion, within public organizations, it is posited that the motivation driving innovation is more strongly associated with PSM than with organizational commitment (Rosa et al., 2020).

Lastly, we discovered that although organizational culture is an important factor, as demonstrated in previous studies (Naveed et al., 2022; Pradana et al., 2022; Budur et al., 2024), its moderating role of organizational culture is not uniformly influenced and varies depending on other individual characteristics, including commitment or motivation. Furthermore, the lack of a significant moderating effect of perceived innovative culture on the PSM-innovative behavior link indicates that the powerful drive of PSM may transcend organizational cultural influences. These findings underscore PSM’s potent and independent role in fostering innovation within the public sector, highlighting the need for further exploration into how motivational dynamics operate in contexts where public service and innovation intersect, without necessarily relying on organizational commitment or culture to facilitate this process.

#### 5.2.2 Practical implications

From a practical standpoint, this study offers actionable strategies for public sector organizations to foster innovation.

First, by recognizing the link between PSM and innovation, public organizations should develop strategies to attract and retain individuals with high PSM. This could involve highlighting service-oriented values in recruitment and promoting a culture that values public service. Although we focused on innovative behavior, PSM has been identified as playing a role in positive outcomes such as job satisfaction, organizational citizenship behavior, and performance within organizations (Ritz et al., 2016). PSM serves as motivation not only for civil servants but also for citizens and employees in private companies. It is widely acknowledged that training programs can cultivate PSM in public organizations. From an innovative standpoint, emphasizing public values and a sense of community through training programs in an organization can directly or indirectly inculcate this motivation within the workforce, thus fostering innovative behavior to enhance public services for citizens.

Second, the role of perceived innovative culture as a catalyst for innovation underscores the necessity for organizations to deliberately foster such a culture. It is critical to create an environment that not only encourages creativity and risk-taking but also deeply values and supports PSM and organizational commitment. Employees are pivotal in driving organizational innovation, crucial for maintaining a competitive advantage and ensuring long-term sustainability (Borins, 2002). Consequently, cultivating a workplace culture or climate that actively encourages employees to embrace and demonstrate innovative behaviors is increasingly becoming an essential strategic approach for organizations (Guo et al., 2023).

Third, the moderating effect of perceived innovative culture on the PSM-commitment relationship implies a need to balance flexibility and innovation with stability and commitment. Organizations should strive for a culture that simultaneously promotes innovation and values the stability brought by committed employees.

In conclusion, this research offers both theoretical and practical insights, enhancing the understanding of how PSM, organizational commitment, and perceived innovative culture interact to drive innovation in the public sector. This underscores the need for a holistic approach to managing public sector organizations, in which individual motivations and organizational culture are aligned to foster innovative behaviors.

## 6 Conclusion

This study seeks to deepen our understanding of the relationships among PSM, organizational commitment, perceived innovative culture, and innovative behavior in public sector organizations. It illuminates how these factors interact and influence each other, contributing significantly to the body of knowledge in organizational psychology and public administration. These findings underscore the pivotal role of PSM in fostering innovative behavior. Furthermore, this study highlights the critical moderating role of perceived innovative culture in this dynamic. This understanding is invaluable for public-sector organizations striving to enhance innovation and adaptability in a rapidly changing global environment.

However, this study has some limitations that should be acknowledged as opportunities for future research. This study’s findings are based on a specific demographic and institutional context, which may limit their generalizability. Different public sector environments, cultural contexts, and organizational structures might yield different results. While this study examined perceived innovative culture as a moderating factor, the multifaceted nature of culture suggests that other cultural elements could influence the observed relationships. Further research could explore additional cultural dimensions and their interplay with PSM and innovative behavior. Other variables not considered in this study may influence innovative behavior in public organizations, such as personal values, leadership styles, policy environments, or external societal pressures (Borins, 2002; Purc and Laguna, 2019). A shortened version of measures was utilized for PSM, organizational commitment, and innovative behavior in this study, potentially compromising the validity of the constructs. Future research should replicate using a full scale of measurement for those variables.

In conclusion, while this study contributes to a valuable understanding of the factors driving innovative behavior in public organizations, it highlights the need for continued exploration in this field. Future research should build on these findings, explore new contexts and incorporate diverse methodologies to further our understanding of innovation in the public sector.

## Data availability statement

The data analyzed in this study is subject to the following licenses/restrictions: The data was created by the Korea Institute of Public Administration and is used with permission in accordance with the Research Data Management Rules of the Korea Institute of Public Administration. The data that support the findings of this study are available on request from Korea Institute of Public Administration. Requests to access these datasets should be directed to https://www.kipa.re.kr/site/kipa/stadb/selectBaseDBFList.do.

## Ethics statement

The studies involving humans were approved by Korea Institute of Public Administration. The studies were conducted in accordance with the local legislation and institutional requirements. The participants provided their written informed consent to participate in this study. Written informed consent was obtained from the individual(s) for the publication of any potentially identifiable images or data included in this article.

## Author contributions

GL: Conceptualization, Data curation, Formal analysis, Funding acquisition, Investigation, Methodology, Project administration, Resources, Software, Supervision, Validation, Visualization, Writing – original draft, Writing – review & editing. CK: Conceptualization, Data curation, Formal analysis, Funding acquisition, Investigation, Methodology, Project administration, Resources, Software, Supervision, Validation, Visualization, Writing – original draft, Writing – review & editing.
